# A Novel Technique for Closed Reduction and Fixation of Paediatric Calcaneal Fracture Dislocation Injuries

**DOI:** 10.1155/2013/928938

**Published:** 2013-05-30

**Authors:** Radwane Faroug, Paul Stirling, Farhan Ali

**Affiliations:** ^1^Department of Paediatric Orthopaedic Surgery, Royal Manchester Children's Hospital, M13 9WL, UK; ^2^University of Manchester, Medical School, Stopford Building, Oxford Road, Manchester, M13 9PT, UK

## Abstract

Paediatric calcaneal fractures are rare injuries usually managed conservatively or with open reduction and internal fixation (ORIF). Closed reduction was previously thought to be impossible, and very few cases are reported in the literature. We report a new technique for closed reduction using Ilizarov half-rings. We report successful closed reduction and screwless fixation of an extra-articular calcaneal fracture dislocation in a 7-year-old boy. Reduction was achieved using two Ilizarov half-ring frames arranged perpendicular to each other, enabling simultaneous application of longitudinal and rotational traction. Anatomical reduction was achieved with restored angles of Bohler and Gissane. Two K-wires were the definitive fixation. Bony union with good functional outcome and minimal pain was achieved at eight-weeks follow up. ORIF of calcaneal fractures provides good functional outcome but is associated with high rates of malunion and postoperative pain. Preservation of the unique soft tissue envelope surrounding the calcaneus reduces the risk of infection. Closed reduction prevents distortion of these tissues and may lead to faster healing and mobilisation. Closed reduction and screwless fixation of paediatric calcaneal fractures is an achievable management option. Our technique has preserved the soft tissue envelope surrounding the calcaneus, has avoided retained metalwork related complications, and has resulted in a good functional outcome.

## 1. Introduction

Calcaneal fractures in children are comparatively rarer than in adults, making up around 0.005% of all fractures reported [1]. Treatment is usually conservative; however, open reduction and internal fixation are often used to manage displaced or intra-articular fractures. We describe the management of an extra-articular calcaneal fracture dislocation using closed reduction with K-wire fixation.

## 2. Case Report

A seven-year-old previously healthy boy was hit by a bus in a 30 mile an hour urban zone. He was taken to the regional paediatric trauma centre where he was managed according to ATLS principles. The secondary survey revealed a closed fracture dislocation of his left calcaneus and bruising over his right hip, which CT scan showed to be an undisplaced fracture of the anterior column of the right acetabulum. Clinically, the calcaneal injury was evidenced by swelling, bruising, and localised tenderness over the left heel. Additional signs included skin puckering on the medial calcaneal aspect and bossing on the lateral side caused by the prominence of the displaced fracture fragment.

**Figure 1:**
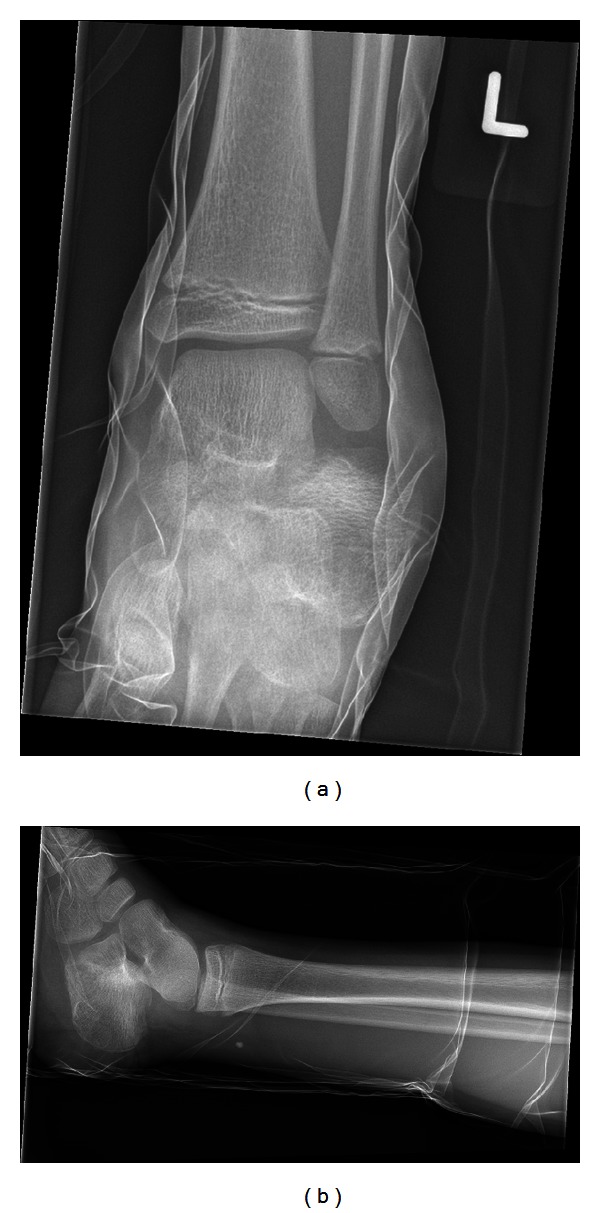
(a) Anteroposterior radiograph displaying two-part extra-articular calcaneal fracture. (b) Lateral radiograph displaying two-part extra-articular calcaneal fracture.

 Anteroposterior and lateral radiographs confirmed an extra-articular two-part calcaneal fracture with proximal and lateral displacement of the posterior calcaneal fracture fragment (Figures 1(a) and 1(b)). 

**Figure 2:**
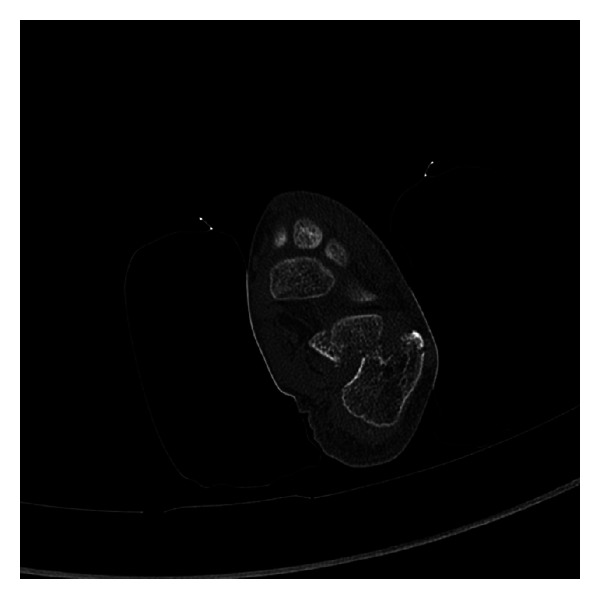
CT scan revealing fracture through body of calcaneus.

**Figure 3:**
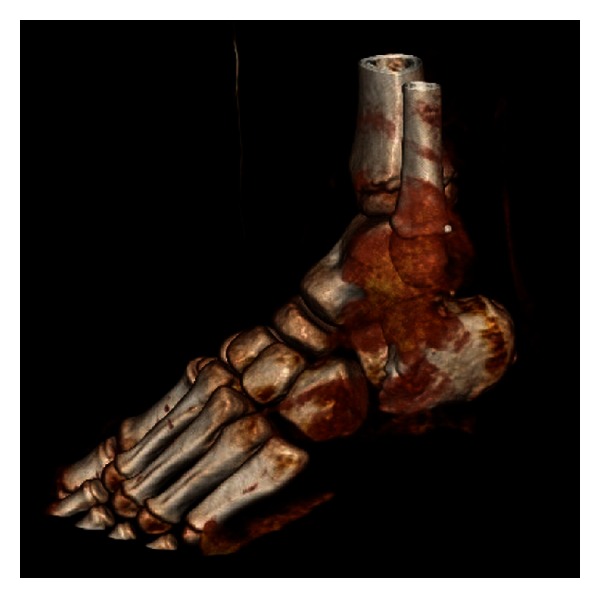
CT 3D reconstruction of left ankle displaying fracture and dislocation through calcaneal body.

CT scan of the foot showed a significantly displaced fracture dislocation through the body of the calcaneus involving the subtalar joint. The ankle mortise and tarsal metatarsals were intact (Figure 2). Computed tomography with 3D reconstruction was used to further evaluate this injury and aid preoperative planning (Figure 3).

Given the soft tissue injury accompanying this injury, it was felt that closed reduction would be of distinct benefit in helping to avoid potential infection and wound healing problems that can complicate open reduction [2, 3].

## 3. Our Technique for Closed Reduction

**Figure 4:**
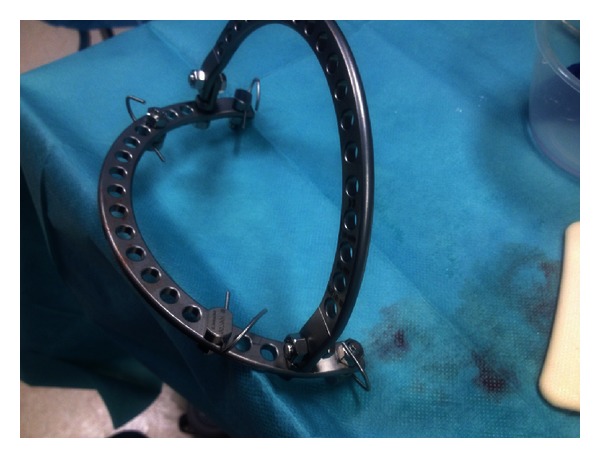
Perpendicular arrangement of Ilizarov half-rings.

In this case, closed reduction was achieved using two Ilizarov half-rings placed perpendicular to each other (Figure 4) and fixed to the calcaneus by way of two 1.6 mm frame wires tensioned to 90 Newtons. Our closed technique consists of two steps. Under image intensifier imaging, the fracture was first reduced with the Ilizarov construct. The second step was maintenance of anatomical reduction using K-wires.

The patient was positioned supine on a radiolucent table. Alcoholic chlorhexidine preparation and adequate draping ensured a sterile field. 

### 3.1. Building the Ilizarov Construct

Two 1.6 mm K-wires were passed through the calcaneus in the axial plane and fixed to one Ilizarov half ring placed around the posterior aspect of the child's heel. One of the two frame wires was placed perpendicular to the posterior calcaneal fracture fragment and drilled medial to lateral. The second frame wire was drilled lateral to medial at an acute angle to the first with a more posterior calcaneal entry point. These wires were locked to the first Ilizarov half-ring and tensioned to 90 Newtons. 

The second Ilizarov half ring was secured perpendicular to the first using nuts and bolts to create an arch over the top of the child's midfoot. The purpose of this was to facilitate controlled reduction in the longitudinal and axial planes.

### 3.2. Reduction and Fixation

**Figure 5:**
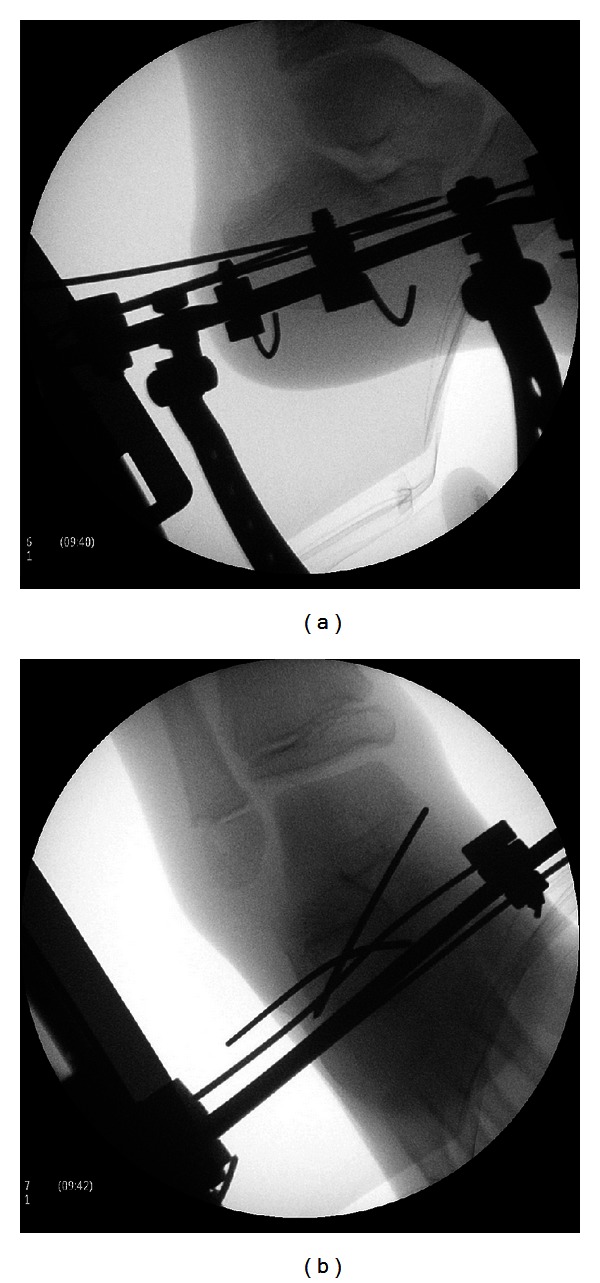
(a) Lateral radiograph confirming anatomical reduction. (b) Anteroposterior radiograph confirming anatomical reduction and demonstrating position of K-wires.

This construct enabled application of longitudinal traction and countertraction, with simultaneous rotational manipulation of the injury. Reduction proved relatively easy and was achieved by combination of longitudinal traction and rotation counter to the deformity. When a palpable clunk was felt, image intensifier Anteroposterior and lateral radiographs confirmed anatomical reduction as verified by restoration of the angles of Bohler and Gissane (Figures 5(a) and 5(b)).

Whilst maintaining reduction, two 2 mm K-wires were drilled posteroanteriorly through the calcaneus to fix the fracture fragments in position. The wire entry points were the posterior aspect of calcaneus and were aimed in the direction of the talus and cuboid, respectively. The wires crossed in the axial plane. Intra-articular wire placement was avoided. The Ilizarov half-ring construct was then removed. 

**Figure 6:**
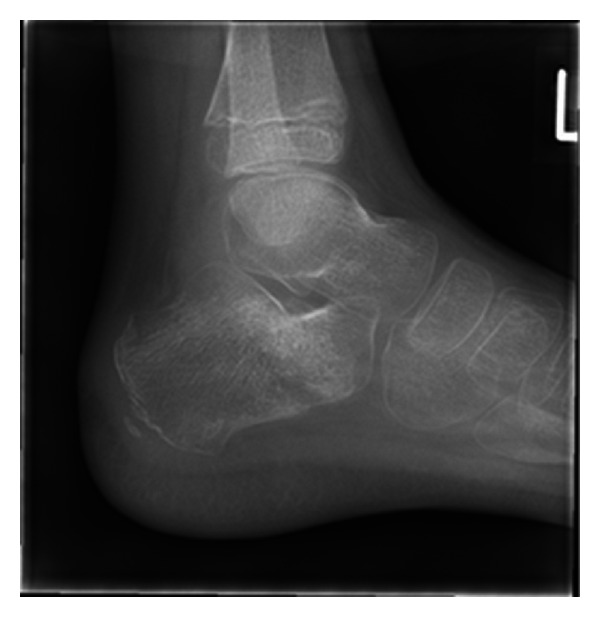
Lateral radiograph at eight-weeks review showing complete bony union.

The patient was followed up over eight weeks until bony union of the injury (Figure 6) when the K-wires were removed in theatre. The patient was fully weight-bearing with a score of 0/10 on the visual analog scale for pain at eight-week review. There were no postoperative complications, and the patient regained full mobility.

## 4. Discussion

Calcaneal fractures in children are usually managed conservatively by immobilisation using a cast or splint, with open reduction and internal fixation reserved for avulsion fractures of the Achilles tendon with displacement of the posterior fracture fragment, or intra-articular fractures [4]. Good postoperative functional outcome after open reduction and internal fixation of paediatric calcaneal fractures has been documented in several case series [5, 6]; however, the low numbers of patients included in these series limits the value of these recommendations. Surgical management of calcaneal fractures in adults is common but has been associated with high rates of complications. Folk et al. reported a series of 190 adult patients treated with open reduction and internal fixation of calcaneal fractures [2], reporting surgical reintervention in 40 cases (21%), of which 4 went on to amputation. Similar rates are reported by Sanders et al. in their series of 120 patients, of which 8 patients developed wound dehiscence, 5 required myocutaneous free flap creation to cover the wounds, and 3 patients required below-knee amputation [3]. By contrast, serious complication rates after open reduction of paediatric calcaneal fractures are much lower [5, 6] than reported in adults, which encourages open reduction for management of these fractures. Open reduction and internal fixation of calcaneal fractures provide good anatomical reduction, essential for management of this inherently unstable fracture, but disturbing the fracture site and periosteum can result in slower healing, malunion, infection, and chronic postoperative pain. Therefore, treatment of closed calcaneal fractures with closed reduction and screwless fixation could result in reduced incidence of pain, infection and malunion, and faster patient mobilisation after injury. Traditional nonoperative management using splints or casts does not disturb the fracture site, and good functional results have been reported in children with extra-articular fractures. Brunet reported a mean AOFAS ankle-hindfoot score of 96.2 (range = 60–90) in a series of 17 patients [7]. Outcomes are poorer for children with intra-articular fractures, however [8]. 

To our knowledge, there are no case series reporting closed reduction of calcaneal fractures in children. Court-Brown et al. reviewed management of two calcaneal fracture dislocations and commented on the injury's irreducibility, labelling the technique of closed reduction as “impossible.” [9] Case series analysing percutaneous reduction with screw fixation [10] or arthroscopically guided minimally invasive techniques [11] for reduction of intra-articular fractures have been reported in adults, but both of these techniques require some degree of soft tissue disruption and screw fixation for adequate reduction and fixation. Use of screws carries with it risks inherent to metalwork retention such as infection and irritation. Preservation of the integrity of the soft tissues surrounding the calcaneus should be the surgeon's main priority in treating this type of fracture [12], as the unique adaptations of the soft tissue envelope which allow efficient weight-bearing can become distorted by surgical reduction, or postoperative infection [13]. This is particularly important given that postoperative infection rates of 11–22% have been reported following open reduction and internal fixation of calcaneal fractures [14–16]. Provided good anatomical reduction is achieved by closed manipulation, and K-wire fixation can maintain this reduction without disturbing the soft tissues surrounding the calcaneus, reducing the risk of postoperative wound infection. This case report illustrates a new surgical technique for closed reduction using Ilizarov frames with K-wire fixation as an effective treatment method for paediatric calcaneal fractures, providing a good functional outcome. Larger series would be required to confirm long-term functional outcomes after closed reduction compared with open reduction. 

## 5. Learning Points

1. Closed reduction and screwless fixation of paediatric calcaneal fractures is an achievable management option.
2. Closed reduction and screwless fixation has in this case preserved the soft tissue envelope surrounding the calcaneus, has avoided retained metalwork related complications, and has resulted in a good functional outcome.
